# Arsenic and Heavy Metal Contamination in Soils under Different Land Use in an Estuary in Northern Vietnam

**DOI:** 10.3390/ijerph13111091

**Published:** 2016-11-05

**Authors:** Thinh Nguyen Van, Akinori Ozaki, Hoang Nguyen Tho, Anh Nguyen Duc, Yen Tran Thi, Kiyoshi Kurosawa

**Affiliations:** 1 Graduate School of Integrated Science for Global Society, Kyushu University, Fukuoka 819-0395, Japan; 2 Institute of Tropical Agriculture, Kyushu University, Fukuoka 812-8581, Japan; a-ozaki@agr.kyushu-u.ac.jp (A.O.); kurosawa@agr.kyushu-u.ac.jp (K.K.); 3 Soil Science Laboratory, Faculty of Land Management, Vietnam National University of Agriculture, Hanoi 100-000, Vietnam; thohoang2211@gmail.com (H.N.T.); nda.khd49@gmail.com (A.N.D.); yenngokhd51@gmail.com (Y.T.T.)

**Keywords:** heavy metals, estuary, soil contamination, spatial distribution, Red River Delta

## Abstract

Heavy metal contamination of soil and sediment in estuaries warrants study because a healthy estuarine environment, including healthy soil, is important in order to achieve ecological balance and good aquaculture production. The Ba Lat estuary of the Red River is the largest estuary in northern Vietnam and is employed in various land uses. However, the heavy metal contamination of its soil has not yet been reported. The following research was conducted to clarify contamination levels, supply sources, and the effect of land use on heavy metal concentrations in the estuary. Soil samples were collected from the top soil layer of the estuary, and their arsenic (As), chromium (Cr), cadmium (Cd), copper (Cu), lead (Pb), and zinc (Zn) concentrations were analyzed, as were other soil properties. Most soils in the estuary were loam, silt loam, or sandy loam. The pH was neutral, and the cation exchange capacity ranged from 3.8 to 20 cmol·kg^−1^. Manganese and iron concentrations averaged 811 µg·g^−1^ and 1.79%, respectively. The magnitude of the soil heavy metal concentrations decreased in the order of Zn > Pb > Cr > Cu > As > Cd. The concentrations were higher in the riverbed and mangrove forest than in other land-use areas. Except for As, the mean heavy metal concentrations were lower than the permissible levels for agricultural soils in Vietnam. The principal component analyses suggested that soil As, Pb, Zn, Cd, and Cu were of anthropogenic origin, whereas Cr was of non-anthropogenic origin. The spatial distribution of concentration with land use indicated that mangrove forests play an important role in preventing the spread of heavy metals to other land uses and in maintaining the estuarine environment.

## 1. Introduction

Heavy metals and metalloids in soil are derived not only from natural sources, such as erosion and the weathering of rocks, but also from anthropogenic sources, such as industrial activities, agricultural production, and household wastewater [1].

Recently, many articles have shown that estuarine aquaculture sediment and agricultural and mangrove forest soil are contaminated by heavy metals [2,3,4,5,6,7,8,9]. The top layer of soil retains heavy metals present in irrigation water through adsorption by soil particles. After soil accumulation, heavy metals can be released into the soil pore water and taken up by plants [10,11]. In aquaculture areas, fish and aquatic organisms can accumulate As, Cu, Pb, Cr, Ni, Cd, and Zn [12,13,14]. Heavy metals in aquatic environments may threaten human health via seafood consumption [13,15,16,17]. Additionally, land uses strongly affect heavy metal and micronutrient distribution in soil [15,18,19]. Therefore, the assessment of heavy metal contamination in estuarine soil (including sediment) is of primary importance.

In the Red River Delta (RRD), the Ba Lat is the biggest estuary in northern Vietnam and has high biodiversity. The mangrove forest of the Ba Lat estuary plays an important role not only in ecological balance but also in the local economy. The mangrove forest environment and ecosystem have deteriorated due to pressures from economic development upstream [2,20,21]. Previous studies in the RRD showed that heavy metals accumulate in the top soil layer of the mangrove forest [20] and that trace metal concentrations vary with land use [22]. There are five main land uses in the estuary, but the heavy metal concentrations of soil under these land uses have not been reported. Furthermore, the relationship between heavy metal concentrations and soil properties (physiochemical properties, soil texture, etc.) has not been clarified.

In the present study, the main purposes were to clarify: (1) the concentration and distribution of heavy metals in soil under different land uses; (2) the relationship between soil properties and heavy metal concentrations; (3) the source of heavy metals; and (4) the effect of land use on heavy metal concentrations.

## 2. Materials and Methods

### 2.1. Study Area 

**Figure 1:**
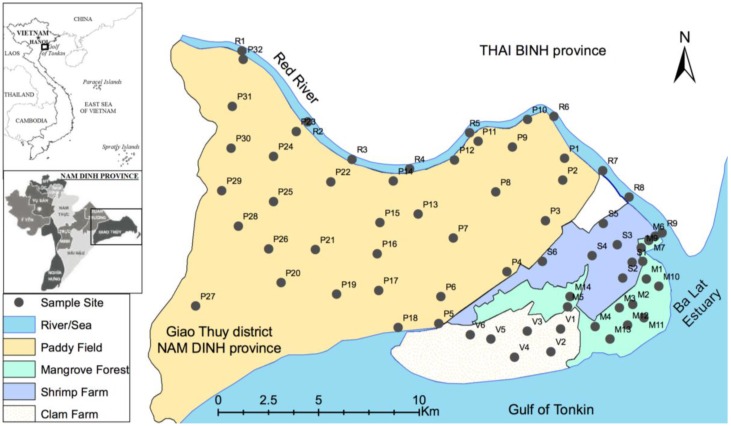
Study area and soil collection sites in the Ba Lat estuary.

In the present study, the Ba Lat estuary, located in the Giao Thuy and Xuan Truong districts of Nam Dinh Province in Vietnam, was targeted. The soils under different land uses were collected from the Ba Lat estuary and their heavy metal concentrations and soil properties were analyzed. The study focused on the following five land uses in this area as shown in Figure 1: (1) paddy fields inside the dike (in the soil sampling section, paddy fields were located inside and outside the dike); (2) extensive shrimp farms; (3) extensive clam farms; (4) mangrove forest located outside the dike, and (5) the river bed of the Red River.

The major soil type in the study area is fluvisol, and rice planting is performed under a developed irrigation system. Irrigation water is taken from the Red River. Many aquaculture farms are located between the dike and the mangrove forest. Among the aquaculture farms, shrimp (*Penaeus monodon*) and clam (*Meretrix meretrix*) farms are targeted because they comprise a large portion of these farms. In shrimp farms but not clam farms, mangrove trees are planted around and inside the farm. Both shrimp and clam farms are affected by daily tides and changes in the water of the Red River.

The dominant trees in the mangrove forests, including Mangrove apple (*Sonneratia caseolaris*), Black mangrove (*Bruguiera gymnorhiza*), Kandelia (*Kandelia candel*), River mangrove (*Aegiceras corniculatum*), and Holy mangrove (*Acanthus ilicifolius*), play an important role in filtering and depositing alluvial material and pollutants transported from upstream [20]. Furthermore, the Ba Lat estuary is an important site for the breeding and stopover of annual migratory birds and provides a habitat for local wildlife.

The estuary is located in a monsoon climate zone with a rainy season from May to October and a dry season from November to April. The annual temperature and rainfall vary from 15.9 to 29 °C and from 1300 mm to 1800 mm, respectively.

### 2.2. Soil Sampling

Soil samples were collected from a total of 67 sites, including each land use. Red River soil (sediment) was collected from the river bed 20 km from the river mouth. The sampling was performed in February 2016. The soil sampling sites are shown in Figure 1.

At each sampling site, top-layer (0–10 cm) soil was collected from five spots in a 10-m^2^ area. A 0.5-kg soil sample was collected at each spot. Samples were packed in plastic bags and transported to the laboratory. Each sample was broken into small pieces, and stones and plants were removed. The sample was dried in a drying oven at 40 °C for 48 h and sieved through a 2-mm sieve. Five soil samples from each site were mixed well, and 500 g of soil was taken from the mixture. The sample was placed in a polyethylene bag and sealed until analysis. Among the 67 samples, 32 samples were collected from paddy fields inside the dike, 26 samples were collected from the field outside the dike, 6 samples were collected from the clam farm, 6 samples were collected from the shrimp farm, 14 samples were collected from the mangrove forest, and 9 samples were collected from the river bed of the Red River. All samples were brought to Kyushu University, Japan, for analysis.

### 2.3. Analytical Methods

#### 2.3.1. Heavy Metal and Soil Property Analyses

Approximately 0.5 g of soil sieved through a 0.2-mm sieve was used for digestion using the United States Environmental Protection Agency (USEPA) 3050B method [23]. The digested solution was filtered through a 4-µm filter (No. 5B; Toyo Roshi Kaisha, Ltd., Tokyo, Japan) and diluted to 100 mL to measure As, Zn, Pb, Cr, Cu, and Cd using inductively coupled plasma-mass spectrometry (ICP-MS, 7500ce, Agilent Technologies, Inc., Santa Clara, CA, USA). The solutions were also used to analyze Fe and Mn using atomic absorption spectroscopy (AAS, Hitachi Z-2300, Hitachi Science & Technology, Tokyo, Japan). Each soil sample was digested and analyzed three times, and their average was used as a representative value.

The organic carbon (OC) content in soil was measured using the Walkley method [24]. The pH was measured using a pH meter (D-55, Horiba Ltd., Kyoto, Japan) and a 1:5 soil/water suspension [25]. The particle-size distribution of the soil was analyzed by the pipette method [26]. The cation exchange capacity (CEC) of the soil was analyzed by the ammonium acetate method [27]. All reagents were of analytical grade, and double-deionized water (Milli-Q Millipore 18.2 MΩ/cm resistivity) was used for dilutions.

#### 2.3.2. Data Analysis

We used R version 3.3.1 [28] on R-Studio version 0.99.902 (RStudio Inc., Boston, MA, USA) [29] for data analysis. The multiple comparison (pairwise comparison) test was applied to determine significant differences in mean heavy metal concentrations of different land uses. The pairwise comparison test was performed using the *stats* package [28].

Principal component analysis (PCA) and other multivariate statistical analysis have been widely applied to identify and apportion contributions between anthropogenic and natural sources [5,6,9,30,31]. To effectively calculate the PCA of similar data, log-ratio transformation is highly recommended to determine and reveal inherent patterns [32,33]. In this study, raw (untransformed) and centered-log-ratio (clr) transformed data [34,35] were used in the PCA. In this study, the principal component analysis was performed using the *FactoMineR* package [36]. The *compositions* package [37] was used to carry out the centered log-ratio transformation. The results of the PCA for both un-transformed and clr-transformed data were visualized using the *factoextra* package [38].

To obtain soil texture classifications, the *ggtern* package [39] was used. To create a box plot of heavy metal data, the *gglot2* package [40] was used. The spatial distribution map of heavy metal concentrations was created using ArcMap 10 software (Environmental Systems Research Institute (ESRI), Redlands, CA, USA).

## 3. Results

### 3.1. Soil Properties in Respective Land Use Areas

Soil properties including pH, cation exchange capacity (CEC), and organic carbon content (OC) that could relate to the absorption ability of heavy metals to soil, soil texture, and total concentration of manganese (Mn) and iron (Fe) are shown in Table 1. According to Table 1, the pH was near neutral,-with a mean of 6.8. Variation in pH was low, with a standard deviation of 0.21.

The organic carbon content ranged from 0.23% to 2.83%, with a mean of 1.66%. According to the mean OC, the OC in land use decreased in the order of paddy field > shrimp farm > mangrove forest > riverbed > clam farm. According to the pairwise comparison test, the OC in the clam farm was significantly lower than in other land uses (*p* value < 0.05), while no significant difference in the OC was observed among the shrimp farm, riverbed, and mangrove forest.

Soil texture in each land use is shown in Table 1. According to Table 1, silt and sand were dominant in the study area. However, the percentages of clay, silt, and sand varied with land use. The average clay percentage in land use decreased in the order of paddy field > shrimp farm > mangrove forest > riverbed > clam farm. The clay percentage was significantly lower in clam farms than in other land uses, while the percentage was significantly higher in the paddy field than in riverbed, clam farm, and mangrove forest (*p* value = 0.05). There were no differences between the other land uses. In addition, the silt percentage significantly differed between each pair.

**Figure 2:**
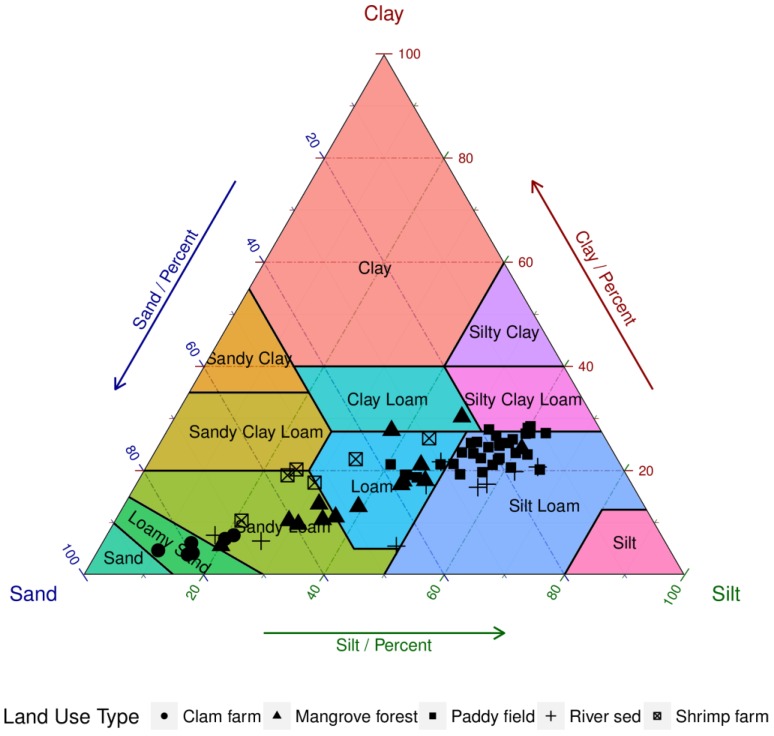
Soil texture of all soil samples based on United States Department of Agriculture (USDA) textural classes.

Figure 2 shows the soil textural class in each land use. Based on Figure 2, the soils were composed mostly of silt loam in the paddy field. In clam farms, the soils were primarily loamy sand and sandy loam. In the mangrove forest and shrimp farm, soils were primarily sandy loam and loam. In the riverbed, soils were mostly silt loam, loam, and sandy loam.

The CEC of soil in each land use is shown in Table 1. The average CECs (cmol·kg^−1^) were 12.8, 12.8, 6.1, 11.6, and 11.9 for riverbed, shrimp farm, clam farm, mangrove forest, and paddy field, respectively. According to the pairwise comparison test, the CEC in the clam farm was significantly lower than in other land uses (*p* value < 0.05). However, the CEC did not differ between paddy field and mangrove forest or between riverbed and shrimp farm.

Table 1 also showed the concentration of Mn and Fe in the soil. According to Table 1, Mn concentration ranged widely, from 189 to 2569 µg·g^−1^, across all samples. Shrimp and clam farms had the lowest average concentration of Mn (516 and 692 µg·g^−1^), whereas mangrove forests had the highest Mn concentration (995 µg·g^−1^). The Fe concentration was high in all samples, ranging from 1.3% to 2.8%. Across the five land uses, the clam farm had the lowest Fe concentration (1.8% on average).

### 3.2. Heavy Metal Concentration of Soil under Different Land Uses

**Figure 3:**
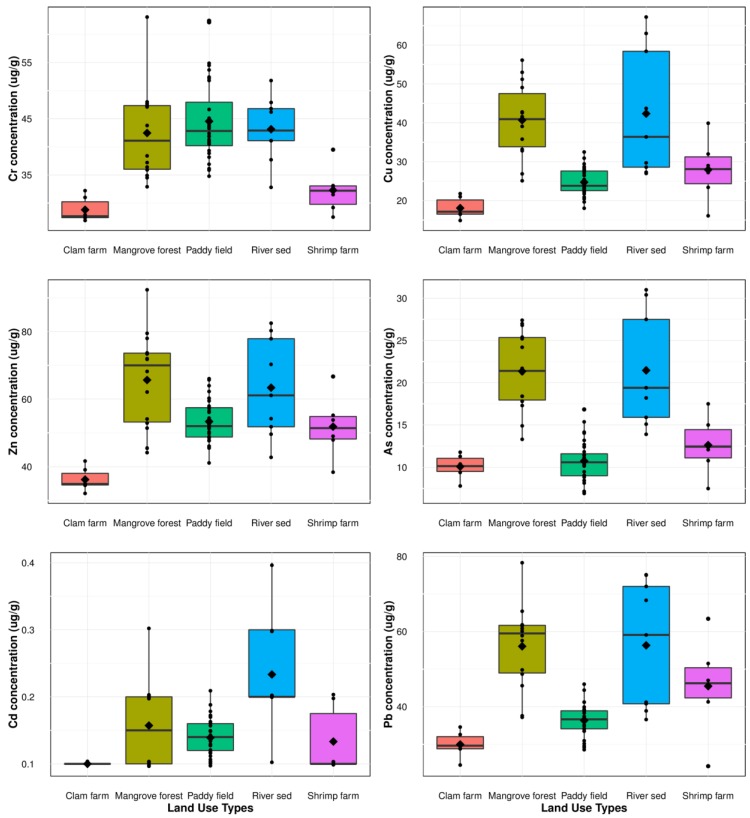
Boxplot of the heavy metal concentrations in soil samples of the five land uses. The black solid line inside the box is the median value; the black rhombus is the mean value; the black dots are individual samples.

The magnitude of soil heavy metal concentrations for all samples decreased in the order of Zn > Pb > Cr > Cu > As > Cd. The heavy metal concentrations for each land use are shown in Figure 3.

Cr concentrations ranged from 26.9 to 63.1 µg·g^−1^, with a wide variation with land use. Soils from clam and shrimp farms had low Cr concentrations (28.8 and 32.3 µg·g^−1^, respectively, on average) that did not significantly differ (Figure 3), while the Cr concentration of the paddy field, mangrove forest, and riverbed were above 40 µg·g^−1^ and did not significantly differ (at *p* value = 0.05) (Figure 3).

Soil Cu concentrations ranged from 14.9 to 67.2 µg·g^−1^. The maximum detected level exceeded the permissible level of 60 µg·g^−1^ for agricultural soil in Vietnam [41]. The average Cu concentration increased in the order of clam farm < paddy field < shrimp farm < mangrove forest < river bed. The Cu concentration in the riverbed varied widely from 27.0 and 67.2 µg·g^−1^, whereas the concentration ranges (µg·g^−1^) were narrow in the paddy field (14.9–21.8) and clam farm (20.8–37.5).

The Zn concentrations (µg·g^−1^) for all samples ranged from 32.1 to 92.4, with a mean of 59.5. All values were within the permissible level for agricultural soil (200 µg·g^−1^). From the viewpoint of land use, the lowest was observed in the clam farm (36.2 µg·g^−1^), while the highest was in the mangrove forest (65.7 µg·g^−1^). The average Zn concentration (µg·g^−1^) was 63.4, 53.3, and 51.8 in the riverbed, paddy field, and shrimp farm, respectively, with no significant differences (at *p* value = 0.05) between them (Figure 3).

The As concentration (µg·g^−1^) for all samples ranged from 6.9 to 31.0, with a mean of 14.5. Most values exceeded the permissible level for agricultural soil in Vietnam (12 µg·g^−1^). In the paddy field, the As concentration exceeded the permissible level at 22% of sites. No sample exceeded the permissible level in the clam farm. The As concentration based on land use decreased in the order of river bed > mangrove forest > shrimp farm > paddy field > clam farm (Figure 3). There was no significant difference (at *p* value = 0.05) among the clam farm, paddy field, and shrimp farm, or between the mangrove forest and river bed (Figure 3).

The Cd concentration (µg·g^−1^) ranged from 0.05 to 0.43, all of which were lower than the permissible level for agricultural soil in Vietnam (2.0 µg·g^−1^). The highest Cd concentration was observed in the riverbed (0.43 µg·g^−1^), while the lowest was observed in the clam farm (0.05 µg·g^−1^).

The Pb concentration (µg·g^−1^) for all samples ranged from 24.2 to 78.3, with a mean of 43.4. Most concentrations were within the permissible level for agricultural soil in Vietnam (70 µg·g^−1^). The Pb concentration based on land use descended in the order of river bed > mangrove forest > shrimp farm > paddy field > clam farm (Figure 3).

### 3.3. Spatial Distribution of Heavy Metals

**Figure 4:**
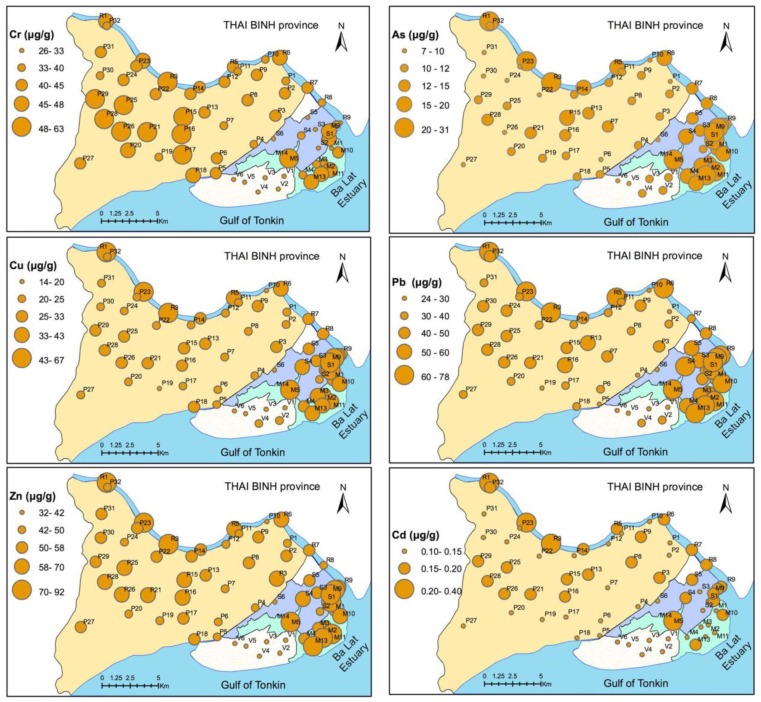
Spatial distribution of heavy metals in the soil of five land uses.

Figure 4 presents the spatial distribution of heavy metal concentrations, in which the bubble represents the level of heavy metal concentrations. 

According to Figure 4, high Cr concentrations (>60 µg·g^−1^) were observed in both paddy fields (P15 and P16 sites) and mangrove forests (M14 site). In contrast, Cr concentrations below 40 µg·g^−1^ were observed in clam and shrimp farms. In the riverbed, samples from the mouth of the river (R8 and R9 sites) had lower concentrations than at other sites.

The Cu, Pb, Zn, As, and Cd concentrations all demonstrated similar spatial distributions. Hotspots were observed in the upstream riverbed (R1, R2, and R3 sites), mangrove forest, and shrimp farm. Heavy metal concentrations, especially Cd concentrations, were low in the clam farm. Concentrations were lower in the paddy field than in the riverbed and mangrove forest, except for Cr. The paddy field near the center (P15, P16, P25, and P29 sites), which had a high population density, tended to have higher concentrations than other sites.

### 3.4. Principal Component Analysis of Heavy Metal Concentrations and Soil Properties

**Figure 5:**
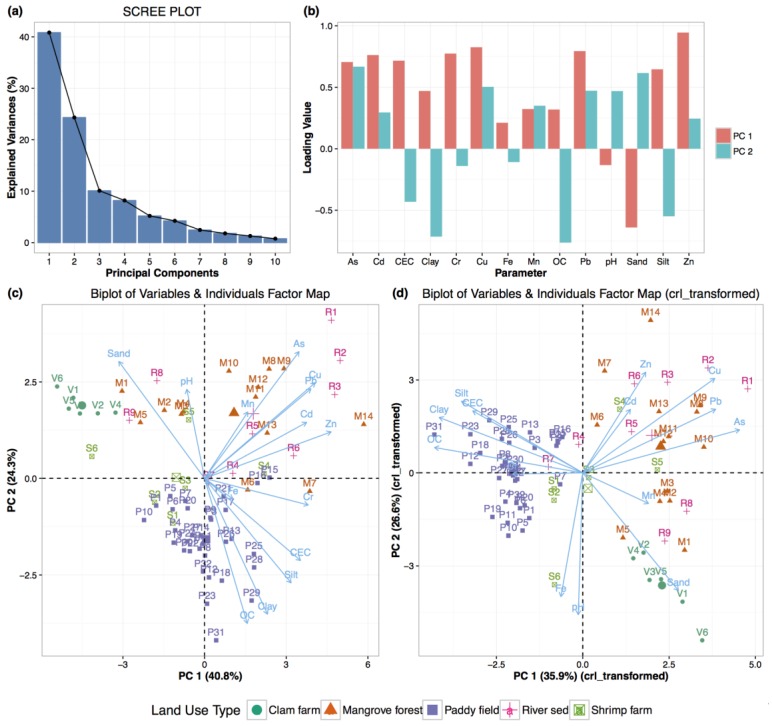
Results of the principal component analysis for all heavy metals and soil properties in five land uses. (**a**) Scree plot for the first 10 components of untransformed data; (**b**) Bar graph of the loading values in the first two principal components of untransformed data; (**c**) The biplot for PC1 and PC2 of untransformed data; (**d**) The biplot for PC1 and PC2 of the centered log ratio (crl) transformed data. PC, principal component.

The results of PCA for all heavy metals, soil properties, and soil samples are displayed in Figure 5. Figure 5a shows values for the first two components that account for 65.2% of the overall variability; specifically, components 1 and 2 accounted for 40.8% and 24.3%, respectively. The loading values of all variables in the first two components are depicted in Figure 5b. The loading values of principal components (PCs) 1 and 2 indicate that heavy metals were dominant in PC1. Some soil properties (CEC, clay content, silt content, OC) also had high loading values in PC1. In contrast, the sand content and pH had negative loading values in PC1. The loading values of all variables in PC2 were divided into negative loading values (CEC, clay, silt, OC, Cr, and Fe) and positive loading values (As, Cd, Cu, Pb, Zn, and Mn).

Figure 5c was plotted based on the coordinates (loading and score values in PC1 and PC2) of 14 parameters and 67 samples extracted from untransformed data. Figure 5d shows a similar plot for the clr-transformed data, explaining 62.5% of the variability. The arrow length (loading value of the variables) is proportional to the variability in PC1 and PC2, and the angle between the two arrows shows the correlation between the variables. In Figure 5c,d, the variables were divided into two groups. The first group is comprised of Cr concentration, CEC, silt, clay, and OC and is located in the lower-right and upper-left quadrants in Figure 5c,d, respectively. In contrast, the second group is comprised of Zn, Cd, Cu, Pb, and As concentrations, located in both the upper-right quadrant of Figure 5c and the upper-right quadrant of Figure 5d. The relationship of the concentrations of Fe and Mn to heavy metal concentrations was weak.

Figure 5c,d display individual scores in PC1 and PC2, where the paddy field is composed of the first-group variables. However, most of the riverbed and mangrove forest data points and some of the shrimp farm data points are composed of second-group variables. Sites R1, R2, R3, M8, M9, and M10 had high scores composed of second-group variables. In contrast, the clam farm is composed of sand content, located in a distinct quadrant with all heavy metals.

## 4. Discussion

### 4.1. Heavy Metal Accumulation and the Effect of Soil Properties in Relation to Land Use

In the clam farm, sand was dominant among soil particles (Table 1). In addition, OC, clay content, and CEC in the farm were lowest across all land uses. The clam farm is located outside the dike (Figure 1) and is affected by tidal and river water-level fluctuations. These fluctuations may work to lower heavy metal concentrations; there was an additional effect from the soil’s low adsorption due to low clay content.

In contrast, the shrimp farm located outside the dike has high values of OM and clay content (Table 1). As mentioned previously, the shrimp farm is built on the site of a former mangrove forest, with mangrove plantations surrounding and inside the farm. These conditions may positively affect the heavy metal concentrations inside the shrimp farm.

The riverbed and mangrove forest had the highest heavy metal concentrations among all land uses. The mangrove forest retains sediment from the Red River due to its location (Figure 1). The dike and controllable irrigation systems not only prevented saltwater intrusion and flooding, but also reduced the heavy metal retention in paddy field. Van Santen et al. [42] showed that the retention of sediment and alluvial materials was nearly 10 times higher in mangrove forests than in the paddy fields in the Red River region. Thus, soil in the mangrove forest has a high potential to retain heavy metals.

### 4.2. Sources of Heavy Metals in Soils

Based on Figure 5c,d, As, Pb, Cu, Zn, and Cd were highly correlated among themselves and were dominant in the same principal component, which suggests that the heavy metals were of anthropogenic origin [4], and in particular, of industrial origin. In contrast, the Cr concentration and CEC, clay, silt, and OC contents, composed of another component, were thought to be associated with the biogeochemical process. This separation of components shows that Cr may not be of anthropogenic but instead may be of non-anthropogenic origin.

According to Figure 4, most heavy metal concentrations, excluding Cr, were higher in the river bed and mangrove forest than in the paddy field. In addition, these concentrations did not change in the mangrove forest and shrimp farm located near the river bed. These characteristics show that heavy metals in the Ba Lat estuary were transported from the upstream portion of the Red River.

Based on the above data, we conclude that heavy metal accumulation varies with land use. The mangrove forest had higher heavy metal concentrations compared to the paddy field and clam farm. This finding indicates that the mangrove forest retained soil that adsorbed heavy metals and prevented soil from flowing out to clam and shrimp farms. Thus, the mangrove forest was found to have an important role in preventing heavy metal contamination of the estuary.

## 5. Conclusions

The following conclusions were derived from this study:

1. High concentrations of As, Pb, Cu, Cd, and Zn were observed in the river bed and mangrove forest. A high Cr concentration was observed in the paddy field. Heavy metal concentrations were lower in the clam farm compared to other land uses.
2. Soil properties of CEC, OC, clay, and silt content were correlated with each other. The concentrations of Cr, Cu, Zn, As, Cd, and Pb were also correlated with each other. Mn and Fe showed a weak relationship with heavy metals and soil properties.
3. The principal components analysis led to two distinct groups of the heavy metals. We associate one of these groups (consisting of As, Pb, Cu, Cd, and Zn) with anthropogenic processes, and the other (consisting of Cr) with non-anthropogenic processes.
4. The mangrove forest plays an important role in preventing the dispersion of heavy metals from upstream to aquaculture areas and the estuarine environment.

## Figures and Tables

**Table 1:** Soil properties of samples from different land uses.

Land Use Type	Parameter	pH	OC	CEC	Soil/Sediment Texture			Mn	Fe
			(%)	(cmol·kg^−1^)	Clay (%)	Silt (%)	Sand (%)	(µg·g^−1^)	(%)
Red River (*n* = 9)	Average	6.87	0.99	12.8	14.8	48.1	37.0	939	1.84
	Max	7.21	1.25	15.8	21.7	65.2	74.3	1495	1.78
	Min	6.51	0.62	7.8	5.5	18.1	14.1	513	2.02
	SD	0.23	0.23	2.7	6.4	16.0	21.2	336	0.07
Shrimp farm (*n* = 6)	Average	6.74	1.45	12.8	19.3	29.8	50.8	516	1.82
	Max	7.03	2.83	16.0	26.2	44.4	68.5	906	1.76
	Min	6.52	0.69	8.2	10.4	21.1	29.4	204	1.91
	SD	0.19	0.77	2.9	5.2	8.4	13.2	285	0.04
Clam farm (*n* = 6)	Average	6.89	0.56	6.1	5.6	16.3	78.1	692	1.55
	Max	7.02	0.94	9.0	7.6	21.2	85.2	845	1.31
	Min	6.75	0.23	3.8	3.9	10.1	71.2	469	1.76
	SD	0.11	0.25	1.8	1.5	4.0	5.2	145	0.21
Mangrove forest (*n* = 14)	Average	6.87	1.09	11.6	16.5	39.3	44.2	995	1.82
	Max	7.03	2.64	20.2	30.3	60.8	74.3	2032	1.76
	Min	6.53	0.70	7.0	5.6	20.1	15.0	320	1.91
	SD	0.14	0.66	3.3	7.3	10.1	16.2	501	0.04
Paddy field (*n* = 32)	Average	6.73	1.66	12.2	24.6	54.2	22.9	772	1.79
	Max	6.98	2.59	19.7	56.5	65.8	76.2	2569	1.48
	Min	6.20	1.18	8.4	18.7	23.8	9.5	189	2.76
	SD	0.23	0.39	2.8	6.7	8.4	13.0	556	0.20
All samples (*n* = 67)	Average	6.80	1.33	11.7	19.4	44.7	36.7	811	1.79
	Max	7.21	7.21	20.2	56.5	65.8	85.2	2569	1.31
	Min	6.20	0.23	3.8	3.9	10.1	9.5	189	2.76
	SD	0.21	0.59	3.3	8.5	15.3	21.7	481	0.17

OC, organic carbon content; CEC, cation exchange capacity; SD: standard deviation.
